# Serum Prestin and Otolin-1 Levels in Pilots of Helicopter-Based Emergency Medical Services: Potential Markers for Ear Injury

**DOI:** 10.7759/cureus.55936

**Published:** 2024-03-11

**Authors:** Piercarlo Minoretti, Andrés Santiago Sáez, Miryam Liaño Riera, Manuel Gómez Serrano, Ángel García Martín

**Affiliations:** 1 Occupational Health, Studio Minoretti, Oggiono, ITA; 2 Legal Medicine, Hospital Clinico San Carlos, Madrid, ESP; 3 Legal Medicine, Psychiatry, and Pathology, Complutense University of Madrid, Madrid, ESP

**Keywords:** inner ear, biomarkers, office workers, construction laborers, helicopter pilots, otolin-1, prestin

## Abstract

Introduction: Occupational noise exposure is a major public health concern, impacting a large workforce worldwide. In this study, we sought to evaluate the serum concentrations of prestin, a cochlear protein that diminishes following noise exposure, and otolin-1, a protein secreted into the bloodstream subsequent to inner ear damage, among three diverse professional categories, each exposed to varying degrees of noise. Helicopter emergency medical service (HEMS) pilots and construction workers were considered high-risk groups due to their elevated exposure to occupational noise, whereas office workers were regarded as a low-risk group, reflecting their comparatively minimal noise exposure.

Methods: The study sample included 60 males, encompassing helicopter pilots, construction laborers, and office workers (n=20, each). Recruitment occurred during standard occupational health visits, with all participants presenting normal clinical audiograms. Serum levels of prestin and otolin-1 were measured in duplicate using commercially available immunoassays and compared across the three professional categories.

Results: HEMS pilots had the lowest mean serum prestin level at 211±27 pg/mL, followed by construction workers at 234±29 pg/mL, and office workers at 269±42 pg/mL (p<0.001, one-way analysis of variance), with all inter-group differences statistically significant (p<0.05, Tukey's post hoc tests). For otolin-1, HEMS pilots showed the highest mean at 216±20 pg/mL, with construction workers at 196±22 pg/mL, and office workers at 181±20 pg/mL (p<0.001, one-way analysis of variance). Statistically significant differences were found between HEMS pilots and both other groups for otolin-1 levels (p<0.05, Tukey's post hoc tests), but not between construction workers and office workers.

Conclusions: Serum concentrations of prestin and otolin-1 may differ among healthy individuals according to their occupational noise exposure and have the potential to act as indicators of subclinical inner ear injury. To substantiate these preliminary observations, incorporating exposure assessment, especially via direct measurements of noise and vibration exposure, would markedly improve the reliability of our findings.

## Introduction

Occupational noise exposure is a significant public health concern that affects a vast number of workers globally [1,2]. In the United States alone, it is estimated that 22 million individuals in the workforce are exposed to noise levels in their workplaces that may be detrimental to their health [3]. Despite the implementation of protective measures and the establishment of regulatory standards [4], approximately one-third of working-age adults with a history of exposure to occupational noise demonstrate audiometric signs indicative of noise-induced hearing damage, with 16% suffering from considerable hearing loss [5]. The consequences of noise-induced hearing loss are profound, as it impairs the ability to detect high-frequency sounds, comprehend speech, and communicate effectively [1,2]. Furthermore, chronic exposure to noise at or above 85 decibels (dB) is linked to a broad range of health issues, encompassing cardiovascular diseases, depressive symptoms, and disorders affecting balance [1,2].

Helicopter emergency medical services (HEMS) pilots are consistently exposed to high levels of occupational noise [6]. Notably, these pilots may experience noise levels with an equivalent continuous sound level ranging from 90 to 95 dB, significantly exceeding the 80-85 dB typically encountered by pilots of fixed-wing aircraft [7]. Similarly, construction workers represent another demographic subjected to substantial occupational noise exposure [8]. Research targeting apprentices in the building trades documented an average daily noise exposure of 87 dB [9]. In addition, it was observed that an increment of 10 dB in noise exposure is associated with a hearing loss of 2-3 dB within the 3-6 kHz frequency range over a span of 10 years [10]. In contrast, office workers are generally exposed to considerably lower levels of noise, highlighting the occupational disparities in noise exposure risks. Notably, an Indian study utilized office workers as a control group for comparison against shipyard workers, who were subjected to daily noise levels in excess of 90 dB [11]. The findings showed that none of the office workers had noise-induced hearing loss, whereas 6% of the shipyard workers did exhibit signs of hearing impairment [11].

In recent years, there has been growing interest in inner ear proteins released into the bloodstream as peripheral biomarkers for research related to the inner ear and human hearing [12]. Within this framework, prestin and otolin-1 have attracted significant research attention. Prestin, a protein found in the outer hair cells (OHCs) of the cochlea, plays a crucial role in human hearing [13,14]. Notably, Parker et al. have recently investigated the correlation between serum prestin protein levels and noise exposure levels in young adults [15]. The authors reported a significant negative correlation between serum prestin levels and mean daily noise exposure, indicating that individuals with lower serum prestin tended to routinely encounter higher noise levels [15]. This has been explained as resulting from noise-induced cochlear injury, with fewer intact OHCs undergoing normal prestin production and turnover [15]. Otolin-1 represents another promising inner ear biomarker [16]. This collagen-like protein functions as a scaffold enabling calcium carbonate deposition into otoconia [17]. Otolin-1 is expressed in the vestibular maculae and cristae, as well as within the tectorial membrane, the marginal cells of the stria vascularis, and Claudius cells [16]. Analogous to prestin, otolin-1 can be identified in human serum and its levels have been observed to increase with age, following otologic surgery, and in individuals experiencing sudden sensorineural hearing loss [16].

In the present study, we sought to compare the serum concentrations of prestin and otolin-1 among healthy individuals from three distinct professional sectors, each with varying levels of exposure to occupational noise. HEMS pilots and construction workers were considered high-risk groups due to their elevated exposure to occupational noise, whereas office workers were regarded as low-risk groups, reflecting their comparatively minimal noise exposure.

## Materials and methods

Study participants

This research is part of a larger endeavor to characterize the biomarker profiles associated with various professional groups [18,19]. The study cohort comprised 60 male individuals from the following three distinct occupational categories: HEMS pilots, construction workers, and office workers, with each category represented by 20 subjects. The decision to include only male participants was informed by the prevailing sex demographics within the HEMS and construction industries. The study subjects were selected during standard occupational health assessments at outpatient care facilities. We excluded individuals with a history of psychiatric, neurological, immune, inflammatory, or infectious disorders, as well as those diagnosed with cancer or who had recently received pharmacological treatment. All participants were in good physical health and exhibited normal auditory function, as confirmed by clinical audiometry. Prior to enrollment, all subjects received detailed information about the research protocol and provided their written informed consent. The study received approval from the local ethics committee (reference number: 2022/12) and was conducted in accordance with the principles outlined in the Declaration of Helsinki.

Quantification of serum prestin and otolin-1 levels

Between 8:00 and 10:00 am, blood samples were collected into tubes designed for serum separation from participants who had fasted overnight. Following a 30-minute period of standing, the samples were subjected to centrifugation at a force of 3000 g for a duration of 10 minutes. Subsequently, the separated serum was divided into smaller portions and stored at a temperature of -20°C to be used in later analyses, ensuring that they were not subjected to repeated cycles of freezing and thawing. Serum levels of prestin and otolin-1 were determined by employing commercial enzyme-linked immunosorbent assay (ELISA) kits (San Diego, CA: MyBioSource Inc.), adhering to the guidelines provided by the manufacturer. To ascertain the concentrations of these proteins, standard curves were generated, and the average fluorescence intensity from each assay well was converted into a concentration value based on the linear segment of the respective standard curve. Each assay was conducted in duplicate, and the average of the two readings was taken. The intra- and inter-assay variability was below 6% and 8%, respectively. In an effort to reduce the possibility of biases in measurement due to knowledge of the participants’ occupational backgrounds, the laboratory staff was kept unaware of such information.

Statistics

Prior to the application of statistical procedures, data were analyzed for normality and variance homogeneity, utilizing the Kolmogorov-Smirnov and Levene's tests, respectively. Given that all variables conformed to a normal distribution, parametric methods were employed for group comparisons. Specifically, a one-way analysis of variance (ANOVA) was applied, with subsequent Tukey's post hoc tests for pairwise comparisons. Continuous data are presented as means and standard deviations, whereas categorical data are expressed in the form of counts and percentages. The chi-square test was used to analyze the frequencies. The relationship between biomarker levels and the general characteristics of the study participants was explored using Pearson's correlation coefficient. Analyses were conducted using the SPSS 20.0 software (Armonk, NY: IBM Corp.), and two-tailed p-values <0.05 were considered to denote statistical significance.

## Results

The three study groups demonstrated no significant differences with regard to age, body mass index, total cholesterol, fasting plasma glucose levels, creatinine, and liver enzymes (Table 1). This indicates a well-balanced distribution between the groups in terms of potential confounding factors. Serum prestin and otolin-1 concentrations for the three professional groups are reported in Table 2.

**Table 1:** General characteristics of the study participants. HEMS: helicopter emergency medical service; AST: aspartate aminotransferase; ALT: alanine aminotransferase; ns: not significant Data are expressed as mean±standard deviation.

Variable	HEMS pilots (n=20)	Construction workers (n=20)	Office workers (n=20)	p-Value
Male	20	20	20	ns
Age (years)	39.1±3.5	38.9±3.4	38.7±2.9	ns
Body mass index (kg/m^2^)	24.1±2.1	23.9±2.3	24.2±1.9	ns
Total cholesterol (mg/dL)	207±12	209±8	211±10	ns
Fasting plasma glucose (mg/dL)	90±11	88±10	91±12	ns
Creatinine (mg/dL)	0.9±0.2	0.9±0.2	0.9±0.2	ns
AST (U/L)	27±12	25±11	26±13	ns
ALT (U/L)	29±13	28±10	30±11	ns

**Table 2:** Serum prestin and otolin-1 concentrations in HEMS pilots, construction workers, and office workers. ^*^P<0.05 versus construction workers. ^**^P<0.05 versus office workers. Data are expressed as mean±standard deviation. HEMS: helicopter emergency medical service

Biomarker	HEMS pilots (n=20)	Construction workers (n=20)	Office workers (n=20)	p-Value
Prestin (pg/mL)	211±27^*,**^	234±29^**^	269±42	<0.001
Otolin-1 (pg/mL)	216±20^*,**^	196±22	181±20	<0.001

**Figure 1:**
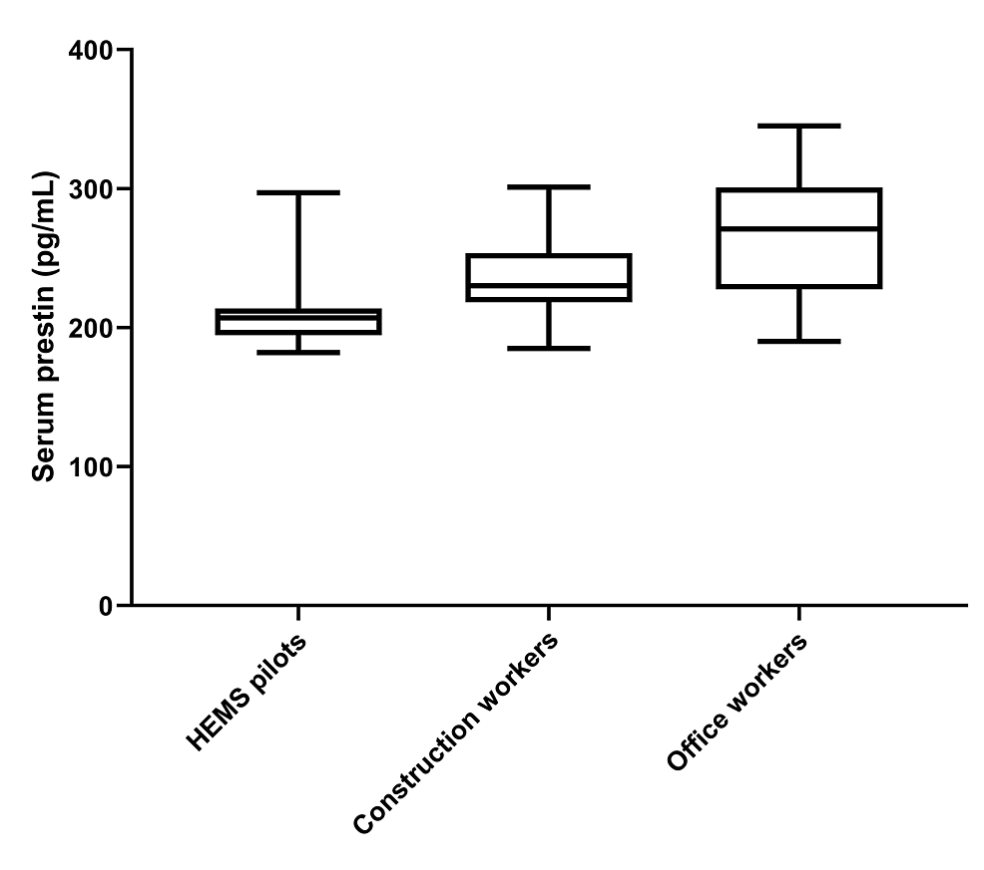
Box plots representing the serum levels (pg/mL) of prestin in the three study groups. HEMS: helicopter emergency medical service

One-way ANOVA demonstrated highly significant inter-group differences in the levels of both prestin and otolin-1 (p<0.001 for both), suggesting pronounced disparities in the concentrations of inner ear-specific biomarkers across the three professional categories. Specifically, HEMS pilots exhibited the lowest mean serum concentration of prestin, recorded at 211±27 pg/mL. This was followed by construction workers, who demonstrated a mean level of 234±29 pg/mL, and office workers, who had a mean concentration of 269±42 pg/mL (p<0.001) (Figure 1). Further analysis through post hoc Tukey's tests revealed that all pairwise comparisons among the three study groups were statistically significant (p<0.05 for all).

**Figure 2:**
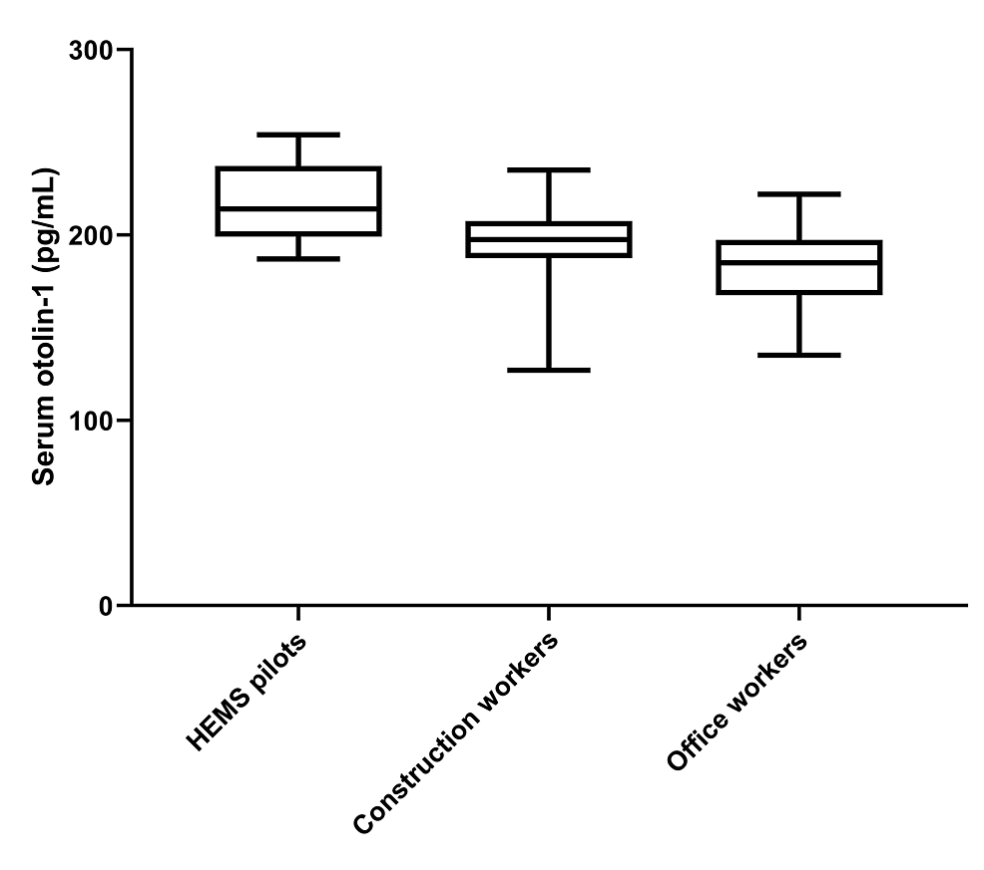
Box plots representing the serum levels (pg/mL) of otolin-1 in the three study groups. HEMS: helicopter emergency medical service

Regarding the serum concentrations of otolin-1, HEMS pilots exhibited the highest mean level, quantified at 216±20 pg/mL. Construction workers were observed to have a slightly lower mean concentration, at 196±22 pg/mL. Office workers presented with the lowest mean levels of otolin-1, averaging 181±20 pg/mL (p<0.001) (Figure 2).

Based on the results of Tukey’s post hoc tests, there were statistically significant differences in serum otolin-1 levels between HEMS pilots and the two other professional groups under investigation (both p<0.05). Conversely, the analysis revealed no statistically significant differences between construction workers and office workers. We did not identify any significant correlations between the serum levels of inner ear-specific biomarkers and the general characteristics of the study participants outlined in Table 1 (data not shown).

## Discussion

The current study, examining inner ear-specific biomarkers among three occupational groups with varying noise exposure risk levels, yielded two key findings. First, HEMS pilots, considered at high risk for occupational noise exposure [6,7], demonstrated the lowest serum prestin levels along with the highest otolin-1 concentrations compared to both construction workers (high risk of occupational noise [8,9]) and office workers (low risk of occupational noise [11]). Second, while construction workers showed significantly different serum prestin levels relative to office workers, their otolin-1 levels did not significantly differ.

Prestin is a motor protein expressed in the OHCs of the cochlea, where it contributes to the sensitivity and selectivity of hearing [13]. Its function is associated with the electromotility of OHCs, allowing these cells to contract and expand in response to electrical stimulation, thereby enhancing the mechanical response of the cochlea to sound [14]. Prior studies have shown that diminished levels of serum prestin are linked to noise-induced trauma in OHCs [13]. Despite all participants in our study presenting normal auditory capabilities, as verified through clinical audiometry, the observation that both HEMS pilots and construction workers exhibited significantly reduced levels of serum prestin underscores the potential for subclinical damage to OHCs, likely attributable to elevated levels of occupational noise exposure. This observation aligns seamlessly with existing research, which posits that noise exposure is a reliable predictor of circulating prestin levels [15]. Such findings can be interpreted to reflect a decrease in the number of intact OHCs capable of normal prestin synthesis and turnover, highlighting the subtle impact of long-term occupational noise exposure on cochlear health [15].

In contrast to prestin, which is instrumental in the amplification of auditory signals, otolin-1 plays a pivotal role in the vestibular system, which is integral to the maintenance of balance and spatial orientation [16]. Specifically, otolin-1 is a component of otoconia - the calcium carbonate crystals found within the utricle and saccule of the inner ear [17]. These otolith organs play a critical role in detecting gravity and linear acceleration. Our study found that otolin-1 levels were significantly higher in HEMS pilots compared to construction workers and office workers. However, otolin-1 levels did not differ between construction workers and office workers. This suggests otolin-1 provides unique insights into occupational exposures and their potential impact on the vestibular system, distinct from prestin.

A potential explanation for the observed elevation in otolin-1 levels among HEMS pilots may be attributed to vibration stress. Within the aviation sector, helicopters are distinguished by their significantly higher levels of vibration [20]. This mechanical oscillation, which moves around a central point in wave-like patterns, facilitates energy transfer [21]. In helicopters, vibrations are transmitted through the fuselage structure, ultimately dissipating through the landing gear suspension onto the ground surface [20,21]. In addition, it is important to note that helicopter pilots experience both localized and whole-body vibrations [21]. Research conducted by Yilmaz and Ila has demonstrated that the vestibulocochlear system can be negatively impacted by whole-body vibrations, independent of auditory noise exposure [22]. Based on these findings, we hypothesize that otolin-1 may primarily function as a biomarker for subclinical vibration-induced damage to the inner ear. This possibility is further corroborated by the observed trend of elevated otolin-1 levels in construction workers relative to office workers, although this difference did not reach statistical significance. In this regard, the documented exposure of construction workers to hand-transmitted vibrations from the use of handheld vibrating tools [23], coupled with the exposure of heavy equipment vehicle drivers to whole-body vibrations, lends credence to the theory that vibration stress may be a contributing factor to inner ear damage, as reflected by elevated otolin-1 levels [24].

While this study highlights the potential applicability of prestin and otolin-1 as biomarkers of subclinical inner ear damage in occupational medicine, it is crucial to recognize the limitations inherent in our research methodology. A notable caveat is the absence of specific auditory and vibration exposure data, which renders our interpretation of the findings somewhat conjectural. The relatively modest sample size further introduces a degree of uncertainty, precluding us from asserting the broad applicability of our results. Additionally, the exclusion of female participants from our study limits the extension of our conclusions to females.

## Conclusions

Serum concentrations of prestin and otolin-1 may differ among healthy individuals according to their occupational roles and have the potential to act as indicators of subclinical inner ear injury within occupational medicine. To substantiate these preliminary observations, incorporating exposure assessment, especially via direct measurements of noise and vibration exposure, would markedly improve the reliability of our findings. Further research is necessary to determine if serum levels of prestin and otolin-1 can longitudinally predict the onset of auditory or vestibular impairments across various professional categories.
